# Infant Developmental Outcomes: Influence of Prenatal Maternal–Fetal Attachment, Adult Attachment, Maternal Well-Being, and Perinatal Loss

**DOI:** 10.3390/ijerph19042433

**Published:** 2022-02-20

**Authors:** Grace Branjerdporn, Pamela Meredith, Trish Wilson, Jennifer Strong

**Affiliations:** 1 Health and Behavioral Sciences, The University of Queensland, Brisbane, QLD 4072, Australia; pmeredith@usc.edu.au (P.M.); j.strong@uq.edu.au (J.S.); 2 Mental Health and Specialist Services, Gold Coast Hospital and Health Service, Gold Coast, QLD 4215, Australia; 3 Mothers, Babies and Women’s Health Services, Mater Hospital, Brisbane, QLD 4101, Australia; twilson2@usc.edu.au; 4 Occupational Therapy, University of the Sunshine Coast, Sunshine Coast, QLD 4556, Australia; 5 Occupational Therapy Department, Royal Brisbane and Women’s Hospital, Brisbane, QLD 4029, Australia

**Keywords:** attachment, infancy, developmental outcome, maternal–fetal attachment, prenatal

## Abstract

Identification of prenatal characteristics that predict later infant development may afford opportunities for early intervention, potentially optimizing childhood development outcomes. The aim of the present study was to examine the effects of selected prenatal factors (maternal–fetal attachment, maternal adult attachment, maternal well-being, and previous perinatal loss) on later infant development. Pregnant women were recruited from two antenatal clinics at one tertiary hospital and asked to complete self-report questionnaires. The Bayley’s Scales of Infant and Toddler Development were then completed one to two years after their baby’s birth. Independent samples *t*-tests, correlational analyses, and multivariate linear regression models were conducted. Results from 40 dyads revealed that more favorable maternal–fetal attachment, more secure/less anxious maternal attachment, and higher maternal well-being predicted maternal reports of infant adaptive behavior regardless of previous perinatal loss. Infants of women without perinatal loss scored higher in external observer-rated cognitive development compared to infants of women with previous perinatal loss. While further research is required, findings indicate that a mother’s well-being and her relationship with her baby during pregnancy contributes to positive perceptions of her infant’s daily living skills. Supporting the parenting of women with perinatal loss is required to, in turn, promote optimal cognitive development in infants.

## 1. Introduction

Infant development, conceptualized in terms of cognition, motor skills, language, socio-emotional skills, and adaptive behavior, lays the foundation for later outcomes in school [1], and even into adulthood [2]. Consistent with the biopsychosocial model [3], a range of prenatal factors related to the infant’s genetic make-up, environment, socio-demographics, obstetric factors and maternal psychology have been identified as influencing infant development [4]. Despite this, several promising factors are under-investigated, and particularly those related to the mother’s mental state, feelings of attachment to the unborn baby and others, and previous relational trauma experienced. A greater understanding of the prenatal predictors of infant development may inform early assessment and interventions adopting family-centered [5], trauma-informed [6] and attachment-based approaches [7] to foster infant development. The aim of this study was to examine the predictive capacity of four prenatal maternal characteristics (maternal–fetal attachment, adult attachment pattern, well-being, previous perinatal loss) on later infant development.

### 1.1. Maternal–Fetal Attachment

The thoughts, feelings, and behaviors that a pregnant woman displays towards her unborn child represents a love relationship known as ‘maternal–fetal attachment’ [8]. As indicated in a systemic review by Branjerdporn et al. [9], maternal–fetal attachment has been shown to influence a range of maternal-reported infant outcomes, such as temperament [10], colic [11,12], and developmental delay [13]. More recently, a Turkish study by Arguz Cildir et al. [14] revealed that maternal–fetal attachment was associated with maternal-reported infant behavioral and emotional competence, and development when infants were 21–31 months old. Similarly, a recent Palestinian study [15] with 12-month old infants showed that maternal–fetal attachment in mothers with significant war trauma predicted maternal-reported infant sensorimotor and fine motor skill development using the Minnesota Child Development Inventory [16]. Given that previous research has not examined infant development based on independent assessors, and instead relied on maternal-report, further research is required that uses both maternal-reported and external observer-rated measures to form a comprehensive infant developmental assessment [17].

### 1.2. Adult Attachment

Another maternal characteristic that may be related to infant development is a mother’s own adult attachment pattern. According to attachment theory [18], individuals develop cognitive, emotional, and behavioral schemas (i.e., internal working models) for understanding themselves and the people around them based on early interactions with parents/caregivers [19], which carry through to adulthood [20]. Adults who received predictable and warm responses from parents/caregivers view themselves as loveable and others as trustworthy, contributing to a secure attachment pattern [21]. In contrast, insecure attachment patterns (avoidant and anxious attachment patterns) may develop if attachment figures are unresponsive or not consistently available in times of need [22]. While avoidantly attached adults are less comfortable in intimate relationships and may refrain from disclosing their feelings to others, anxiously attached adults desire intimacy in relationships but fear rejection from others [23].

Alhusen, Hayat and Gross [13] examined cross-sectional associations between adult attachment patterns measured postnatally and maternal-reported infant developmental delays using the Ages and Stages Questionnaire [24]. These authors identified that higher levels of postnatal maternal avoidant and anxious attachment were both associated with delays in overall infant development, as perceived by the mother. While evidence suggests that the mothers’ postnatal adult attachment pattern has a bearing on infant development [13], to the authors’ knowledge, no studies have examined the relationship between prenatal adult attachment and infant development.

### 1.3. Perinatal Loss

Perinatal loss occurs in approximately 18.4 per 1000 births [25]. Immediately following the death of a baby, women may experience intense levels of grief and bereavement [26]. The negative emotional sequelae may extend to the subsequent pregnancy [27], with some women experiencing delays attaching to the subsequent baby, and feeling anxious and hypervigilant about the health of the new baby [28].

To the authors’ knowledge, only one longitudinal study has compared children born from mothers with and without perinatal loss on any outcome, examining infants at 12 months old in relation to security of attachment [29], and again at six to eight years old to investigate emotional and behavioral difficulties, cognitive performance, and child health [30]. Results revealed that infants from mothers with perinatal loss had higher disorganized attachment at 12 months old [29]. At six to eight years old, women with perinatal loss tended to perceive that their children had more difficulties, particularly in relation to peer interactions, compared to women without perinatal loss [30]. Further research is needed regarding the impact of previous perinatal loss on development.

### 1.4. Prenatal Maternal Mental Health 

The prenatal period is a high-risk time for first onset and relapse of anxiety and depression [31]. An association between maternal distress during pregnancy and later suboptimal socio-emotional infant development has been well-recognized [32]. Plausible reasons for this are the fetal programming through increased maternal cortisol levels, and lower infant vagal activity [33]. The effect of prenatal mental health on infant development outcomes has been identified in a variety of specific populations families [34,35], but none for the effects of prenatal mental health for women with and without previous perinatal loss. 

### 1.5. Aims and Hypothesis

The aim of this study is to examine the influence of prenatal maternal–fetal attachment, maternal adult attachment, perinatal loss, and maternal mental health on maternal-rated and observer-rated infant development in the domains of cognition, motor skills, language, adaptive behavior, and social-emotional skills. Based on the available literature, it is hypothesized that lower scores on maternal–fetal attachment, insecure antenatal maternal attachment patterns, higher levels of prenatal maternal distress, and previous perinatal loss will predict lower infant development outcomes. Evidence in support of these hypotheses would afford justification and direction for provision of early intervention services.

## 2. Materials and Methods

### 2.1. Participants 

Pregnant women were eligible to participate if they attended one of two antenatal clinics at a public hospital in Australia: (1) a specialized clinic for those who had previously experienced perinatal loss in their most recent pregnancy (stillbirth after 28 weeks gestation to neonatal death one month postpartum) [36]; and (2) a general antenatal clinic for women receiving standard care. Women with gestational diabetes, who were from a refugee background, or who identified as Aboriginal and Torres Strait Islander were admitted to other clinics at this hospital and were therefore not recruited. Women also had to be over 18 years old and have sufficient English abilities to complete the questionnaire. Following birth, mother–infant dyads were eligible to continue to participate if the infant had no known developmental disabilities (e.g., cerebral palsy, Down Syndrome).

### 2.2. Procedure

A prospective cohort study was completed, with data at other timepoints published [37,38,39]. During pregnancy (Time 1), flyers advertising the study were visible in the waiting rooms of antenatal clinics, and women were approached by one research nurse, independent of clinical care, who explained the study. The research nurse had a standardized script and explained that participation in the study was voluntary and non-participation did not affect clinical care. Detained records relating to the number of women who declined to participate were not kept, but non-participation generally related to the amount of time required and lack of incentive provided. Once informed consent was obtained, women completed self-report questionnaires (Maternal Antenatal Attachment Scale, Mental Health Inventory-38, and Attachment Styles Questionnaire) at a time and place convenient for them. Reply-paid envelopes were provided for women who elected to complete the questionnaires at home.

After infants turned 12 months of age (Time 2), women were sent a letter inviting participation in an infant developmental assessment which consisted of a parent-report survey and an observer-rated assessment. The parent-report survey was sent by mail to the women’s homes with a reply-paid envelope. Again, records relating to the number of women who declined to participate were not kept. For the observational developmental assessment, women were asked to attend a one-hour appointment at the public hospital and were provided a free parking voucher. After the assessment, women were sent a brief infant development report summarizing the scores from the infant developmental assessment. The observational developmental assessment was conducted by a member of the research team (GB) who was an experienced pediatric occupational therapist with knowledge of developmental psychology and trained in the use of the assessment through online modules administered by the assessment provider and work-shadowing of other therapists.

### 2.3. Measures

#### 2.3.1. Demographic Information

At Time 1, women without perinatal loss completed a demographic questionnaire including variables such as marital status, ethnicity, mental health diagnosis, date of birth, and post code. For women with perinatal loss, demographic information in hospital records were extracted by a hospital administration officer to reduce the burden on participants with having to provide this data, as this was considered a more vulnerable population. To represent participants’ social-economic status, the Index of Relative Socio-economic Advantage and Disadvantage (IRSAD) percentile rank within Australia [40], based on area zip code, was used.

#### 2.3.2. Maternal Antenatal Attachment Scale (MAAS)

At Time 1, the relationship between the pregnant woman and her unborn child was measured using the Maternal Antenatal Attachment Scale [8]. The 19-items in the instrument are aggregated into two subscales: *quality of attachment*, evaluating the nature of cognitions and feelings towards the unborn baby, and *time spent in attachment*, assessing the frequency of thoughts and behaviors which represent affection and care towards the unborn baby. Items are rated on a five-point scale (1 = very negative, to 5 = very positive; or 1 = not at all, to 5 = almost all the time). The MAAS has previously demonstrated adequate Cronbach alpha scores (quality of attachment = 0.769, time spent in attachment = 0.745) [37].

#### 2.3.3. Attachment Style Questionnaire (ASQ)

To measure the mother’s adult attachment pattern in general relationships, the Attachment Style Questionnaire (ASQ) [41] was used. This 40-item self-report measure reflects the dimensional nature of adult attachment and was developed with an Australian sample. The tool is comprised of 40 items which are rated on a six-point Likert scale (1 = ‘totally disagree’, 6 = ‘totally agree’). Scores were summed to obtain three subscales: attachment security, and two measures of attachment insecurity (attachment avoidance and attachment anxiety). *Attachment security* assesses an individual’s confidence in self and others in the context of relationship (e.g., ‘I feel confident that other people will be there for me when I need them’). *Attachment avoidance* is a measure of a person’s degree of discomfort in trusting and becoming emotionally close to others, and desire to prioritize independence to avoid vulnerability (e.g., ‘I find it difficult to depend on others’). *Attachment anxiety* represents one’s need for affirmation by others, and anxiety in depending on others (e.g., ‘It’s important to me that others like me’). The total score of each of the subscales were between 13 and 71 (SD = 5.6–11.5). Internal consistency scores, indicated by Cronbach alpha scores, have been high in other studies (attachment security = 0.854, attachment avoidance = 0.894, attachment anxiety = 0.865) [37].

#### 2.3.4. Mental Health Inventory-38 (MHI-38)

The Mental Health Inventory-38 (MHI-38) [42] was used to assess mental health status. This self-report tool consists of 38 items which are mainly scored on a six-point scale (1 = ‘none of the time’, ‘very dissatisfied, unhappy most of the time’; 6 = ‘all of the time’, ‘extremely happy, could not have been more satisfied or pleased’). Two of the thirty-eight items (i.e., items 9 and 28) are scored on a five-point scale (e.g., 1 = ‘yes, to the point that I did not care about anything for days at a time’; 5 = ‘no, never felt depressed at all’). The items may be aggregated into one total score (*mental health index*), with higher scores indicating more positive mental health outcomes (i.e., psychological well-being) and lower scores representing more negative mental health (i.e., psychological distress). The MHI-38 is reported to have internal consistency scores (mental health index Cronbach’s alpha = 0.95) in pregnant women [38].

#### 2.3.5. Bayley Scales of Infant and Toddler Development—Third Edition (Bayley-III)

The Bayley Sales of Infant and Toddler Development—Third Edition (Bayley-III) [43] is considered the ‘gold standard’ for assessing the developmental functioning of infants and young children between 1 and 42 months. The standardized instrument consists of five domains: social-emotional, adaptive behavior, cognition, language, and motor. While the social-emotional and adaptive behavior domains are measured by a parent/caregiver-report questionnaire, the cognitive, language and motor domains are assessed by a trained rater through observation of the infant/child’s performance during specific tasks.

The *social-emotional* component of the survey measures the emotional regulation and interpersonal skills of the infant (e.g., you can help your child to calm down, copies or imitates familiar make-believe play), and has between 11–35 items depending on the age of the child. The *adaptive behavior* component of the questionnaire has 241 items, with the global adaptive composite score measuring the ten daily living skill areas that an infant displays in a variety of contexts. This includes communication (e.g., repeats words others say), community use (e.g., recognizes own home), functional pre-academics (e.g., points to pictures when asked), home living (e.g., points to place where his or her clothes are store), health and safety (e.g., cries or whimpers when he or she does not feel well or is injured), leisure (e.g., plays with a single toy or game for at least one minute), self-care (e.g., holds and drinks from a sipping cup), self-direction (e.g., shows interest in a toy or other object by looking at it for a few seconds), social (e.g., smiles when he or she sees parent), and motor (e.g., walks without help) [43]. The domain of *cognition* measures an infant’s problem-solving and executive functioning skills (e.g., child places at least one peg two or more times in the same or different holes, child attends to entire story). The *language* domain assesses both receptive communication (e.g., child correctly responds to at least two directions with the doll or bear, child correctly identifies at least three clothing items) and expressive communication (e.g., child uses at least eight different words appropriately, child correctly names at least five pictures). The *motor* domain explores both fine motor skills (e.g., child spontaneously and purposely scribbles on paper, child places ten pellets in bottle in 60 s or less one pellet at a time) and gross motor skills (e.g., child balances on left foot while you hold one of his or her hands, child jumps to floor).

For the observational assessment, the rater begins administering tasks based on the adjusted age of the infant. Infants must successfully complete three consecutive items at the start point for any age to proceed onwards; otherwise, the assessor administers tasks for the start point of the previous age. The rater stops administration for the domain once the child has not been able to successfully complete five consecutive items. For each of the domains of development, raw data scores are converted to composite scores based on each age group, which are then converted to percentile scores for comparison with normative data. While there may be subscales within each domain (e.g., fine and gross motor within motor), the percentile rank of the total score for each domain was used. The Bayley-III was standardized on 1700 American [43] infants, and demonstrates acceptable validity and reliability in an Australian sample [44,45].

### 2.4. Statistical Analysis

Data were analyzed using Version 25 of Statistical Package for Social Sciences (SPSS) (IBM, Chicago, IL, USA), with significance levels set at *p* = 0.05 as this was an exploratory study. Based on power calculations using Cohen [46], the sample size at that completed both timepoints (N = 40) was sufficient to detect a large effect size when *p*-value = 0.05. Descriptive statistics for all variables, and Cronbach’s alpha values for relevant subscales, were calculated. Women with and without a history of perinatal loss were compared on study and demographic variables using independent sample *t*-tests and chi-square tests (see Table 1 and Table 2). Data were checked for assumptions prior to conducting correlational and multiple multivariate linear regression analyses. Correlational analyses between independent and dependent variables were conducted. Initially, multivariate linear regression models were conducted with all or multiple combinations of study variables (maternal–fetal attachment variables, maternal adult attachment, maternal mental health) and infant development outcomes that were significant in correlational analyses. To avoid multicollinearity based on the variance inflation factor, separate multiple linear regression models containing individual predictive variables and control variables were completed. Residuals were checked for normality.

## 3. Results

### 3.1. Demographic Characteristics for Women with and without Previous Perinatal Loss

While 108 women completed the Maternal Antenatal Attachment Scale at Time 1, infant developmental data on 40 dyads (37.04%) were collected postnatally, resulting in an attrition rate of 62.96%. There were, however, no differences in demographic characteristics or study variables between women who completed Time 1 only and those who completed both time points.

The demographic characteristics and subscale responses of women with (*n* = 15) and without perinatal loss (*n* = 25) who participated in both Time 1 and Time 2 are outlined in Table 1. Women with and without perinatal loss were similar in that most were in a relationship, from a Caucasian background, and had never received a mental health diagnosis. On average, women were approximately 30 years old, resided in middle-class suburbs, completed questionnaires in the third trimester, and had their infants assessed at Time 2 at about 18–19 months old. While all women with perinatal loss had no other living children, women without perinatal loss had slightly more living children.

Of the women with perinatal loss, 14 women (93.33%) had experienced one perinatal loss, whereas one woman (6.67%) had had two previous losses. The perinatal loss for most women (*n* = 14, 93.33%) was a singleton baby, with one mother (6.67%) losing twin babies. Women, on average, had lost their previous baby at 31.59 weeks’ gestation (SD = 8.41, range = 19.5–42.3 weeks). On average, women gave birth to their subsequent baby 947.73 days (SD = 529.97, range = 445–1967 days) after the most recent perinatal loss.

Adequate internal consistency scores were revealed, with Cronbach’s alpha scores above 0.70 for all MHI-38, MAAS and ASQ subscales (see Table 2). Subscale responses between the two groups were similar on all study variables, including MHI-38, MAAS, ASQ, and most subscales within Bayley-III. The exception was for cognition, whereby infants of women with perinatal loss had significantly lower scores.

### 3.2. Correlations

Pearson correlation analyses were conducted between variables (Table 3). Mental well-being (MHI-38 mental health index) was positively correlated with MAAS quality of prenatal attachment, ASQ attachment security, and Bayley-III adaptive behavior. MAAS quality of attachment was positively correlated with MAAS time spent in attachment and Bayley-III adaptive behavior. ASQ attachment security was positively correlated with Bayley-III adaptive behavior. ASQ attachment anxiety was negatively correlated with mental well-being (MHI-38 mental health index) and Bayley-III adaptive behavior.

When examining correlations between Bayley-III subscales, the social-emotional subscale was positively correlated with the language subscale. Adaptive behavior was positively correlated with the social-emotional subscale. Bayley-III cognition, language and motor subscales were positively and highly correlated with each other.

### 3.3. Multiple Multivariate Linear Regression Modelling

Given that the Bayley-III adaptive behavior subscale was the only domain that was significantly correlated with multiple study variables, multivariate models were conducted with Bayley-III adaptive behavior as the outcome variable. When all or multiple combinations of predictors were placed in the same model, multicollinearity was high. To avoid multicollinearity, models were conducted with individual predictors, controlling for perinatal loss as the only significant demographic characteristic during preliminary analyses.

As shown in Table 4, higher MHI-38 mental health index was associated with increased Bayley-III adaptive behavior. More optimal MAAS quality of attachment significantly predicted higher Bayley-III adaptive behavior. Higher ASQ attachment security was associated with more favourable Bayley-III adaptive behavior. In contrast, reduced ASQ attachment anxiety was associated with decreased Bayley-III adaptive behavior. Perinatal loss was not significant in any of the models.

## 4. Discussion

This study is the first to investigate the association between four prenatal maternal factors (prenatal maternal–fetal attachment, maternal adult attachment, perinatal loss, and distress) and later infant development outcomes. Results extend the current literature by using both maternal-reported and external-rated measures of infant development. The results partially support hypotheses, in that more favourable maternal–fetal attachment, maternal adult attachment, and maternal mental health were associated with more positive infant adaptive behavior outcomes. Maternal–fetal attachment, maternal adult attachment, and maternal mental health were not associated with infant cognition, motor, language, and social-emotional developmental domains. Based on observational developmental assessments, women with perinatal loss tended to have infants who scored lower in cognitive development compared to infants of women without perinatal loss. The findings of the present study relevant to each of the four predictors are discussed below for each of the study predictors.

### 4.1. Maternal–Fetal Attachment

Women who reported higher quality of maternal–fetal attachment (i.e., more warmth, seeing the unborn baby as a person rather than an object) during pregnancy, were also more likely to report that their infants had more optimal adaptive behavioral outcomes when assessed after the age of one year. Maternal–fetal attachment, however, did not predict infant cognitive, language, and motor development when assessed via an external rater at the same time. This suggests that the quality of the maternal–fetal attachment relationship may affect a mother’s perception of her infant’s development (adaptive behavior), although more research is needed to support this contention.

While the association between maternal–fetal attachment and infant adaptive behavior has not previously been studied, this finding corroborates other research whereby poorer maternal–fetal attachment was associated with maternal-rated infant outcomes such as global developmental delay [13], less optimal infant temperament [10], colic [11], and decreased sleep [12]. In contrast with a study by Arguz Cildir, Ozbek, Topuzoglu, Orcin and Janbakhishov [14], in which maternal–fetal attachment was associated with infant/child behavioral and emotional competence, our study revealed no significant association between attachment and the social-emotional scale. While both studies used maternal-report, infants in the present study were slightly younger, and different infant developmental assessments were used; however, further research is required.

### 4.2. Adult Attachment

When examining maternal adult attachment, results of the present study suggest that mothers who reported more secure attachment (i.e., see themselves as worthwhile, confident in other’s availability, find it relatively easy to get close to others) in the prenatal period were more likely to report that their infant exhibited more favorable adaptive behavior outcomes later on. In contrast, pregnant women who were more anxiously attached (i.e., worried that others would not care about them, worried they would not measure up to other people) tended to report that their infant had less optimal adaptive behavioral outcomes. This suggests that the internal working model of a mother either shapes the perception or actual adaptive behavior skills in an infant, with more positive internal working models of self and others leading to more favourable daily living skills for infants.

While our study measured adult attachment prenatally, findings are somewhat congruent with a postnatal study reporting cross-sectional findings between adult attachment and infant developmental delay [13]. Although scores on attachment security were not reported, Alhusen, Hayat and Gross [13] similarly identified that postnatal anxious attachment patterns were associated with maternal-rated global infant developmental delay at one to two years postpartum. This same study [13], however, revealed a significant association between attachment avoidance and global infant developmental delay. The present study found contrasting results in that attachment avoidance was not associated with any of the infant developmental domains such as cognition, motor, language, social-emotional skills, and adaptive behavior. Differences in results for attachment avoidance may be attributed to the differences in population examined with the present study exploring predominantly middle-class Caucasian women in metropolitan Australia, whereas Alhusen et al. [13] examined African American women from low-income backgrounds. An environment of urban poverty, with its related stressors (e.g., exposure to neighborhood crime and violence, housing instability, and lack of employment opportunities) may engender mistrust in others and chronic hypervigilance, and in turn, make it more difficult to nurture and support an infant’s development [47].

### 4.3. Perinatal Loss

Independent assessment of infant cognition revealed that offspring of women with perinatal loss scored statistically significantly lower compared to offspring of women without perinatal loss. This is the first study to examine cognitive outcomes of infants of women and without perinatal loss. When exploring the literature more broadly, Turton, Badenhorst, Pawlby, White and Hughes [30] showed contrasting results in that there were no differences in the intelligence quotient (IQ) of older-aged (aged six to eight years) children born from women with and without perinatal loss. Despite this, Turton, Badenhorst, Pawlby, White and Hughes [30] revealed that women with perinatal loss responded to their children with higher levels of criticism of children’s actions, more controlling behavior, less harmonious emotional atmosphere, and reduced maternal engagement, compared to women without perinatal loss. These suboptimal maternal–child interactions, if present during infancy, may plausibly affect infant cognitive development, which may consequently be a possible mechanism for the present study’s finding. While further research is required to explain this finding, theoretical concepts of the ‘replacement child’ (i.e., where the next child receives parental projections and denigration when compared to the idealized lost child) [48] and the ‘vulnerable child syndrome’ (i.e., where the child is seen as fragile and prone to harm due to death of the previous child) [49] have been used by researchers to elucidate the less optimal outcomes of children of women with perinatal loss.

### 4.4. Prenatal Mental Health

In the present study, women with higher levels of distress in the prenatal period scored their infants lower on their adaptive behavior abilities. A large body of literature has similarly revealed links between prenatal maternal distress and poorer child developmental outcomes [32,50]. In contrast, prenatal maternal distress was not linked with infant social-emotional, cognitive, motor, or language outcomes in the present study. While further research is required to understand the lack of significant relationships between these variables, it is possible that the women in our sample had other protective factors which may have buffered the effects of prenatal distress, such as most not having a history of or a current mental health diagnosis [51], increased social support of being married [52], higher socio-economic status [53], primiparous status [54], and receiving support from a specialized hospital clinic for women who have previously experienced perinatal loss [36].

### 4.5. Limitations

While results are in line with theoretical expectations, they are preliminary and methodological factors limit conclusions. There was a high rate of attrition from Timepoint 1 to Timepoint 2, which may have been due to the lack of incentive and amount of time required with both a face-to-face visit and completion of questionnaires. Due to the relatively small sample size, there is the possibility of Type II errors. Larger studies are required to improve confidence in the results and generalization to different samples. An additional limitation was that the number of women approached at Timepoint 1 and contacted at Timepoint 2 was not recorded. While infant development was measured through both objective assessment and maternal self-report, prenatal data were collected using maternal-report questionnaires, increasing the risk of findings being attributable to shared method variance. Despite this, data were collected over two time points which enabled the present study to propose associations of causality. Selection bias may have occurred as participation in the longitudinal study was voluntary and there was a large drop-out rate between the two time points, potentially limiting the representativeness of the sample. Another limitation was that the external rater conducting the Bayley-III observational assessment and generating the developmental reports was not blind to the participant’s perinatal loss status. While patterns of association between infant developmental domains and maternal distress, maternal–fetal attachment, and adult attachment differed based on whether the infant developmental domains were assessed by the external rater or mother, it is unclear if these differences were due to who the rater was as the same subscales were not assessed. Future studies should obtain reports of the same measures from various parent/caregiver and observer assessors to specifically address the question of maternal perception. Another limitation was that associations between infant development and variables relating to the gestational age of previous perinatal loss, or the other parent/caregivers, were not examined. Participants were drawn from a single site from within Australia, which may limit the generalizability of the results. While it was identified that infants of women without perinatal loss scored higher in cognitive skills compared to infants of women with perinatal loss, the average score for infants of women with perinatal loss was still within the average range. While self-reported measures reveal the participant’s perspective, given that assessment of maternal–fetal attachment was subjectively reported by the mother, the conclusions drawn are to be interpreted with caution. As women from the private sector were excluded from the present study and may be plausibly different, further research is required which explores this population. Given that women completed the prenatal questionnaires both at home and in the hospital, there may be possible bias based on the survey completion setting.

## 5. Conclusions

The present study is the first to investigate whether prenatal maternal–fetal attachment, maternal adult attachment, and maternal mental well-being may predict infant development outcomes in two groups of women, with and without perinatal loss. A novel aspect of the present study is that infant development was examined by an external rater (in the domains of cognition, motor, and language) and maternal-report (in the domains of adaptive behavior and social-emotional development), with all earlier studies using maternal-report questionnaires when examining the relationship between maternal–fetal attachment and adult attachment with infant development. While further research is needed to strengthen confidence in the findings, more optimal maternal–fetal attachment, maternal secure attachment patterns, and well-being supports the development and/or maternal perception of infant daily living skills and activities. This study reveals the potential value of intervening in the prenatal period to support maternal perceptions of infant development by promoting maternal–fetal attachment, secure attachment, and well-being. The study also highlights that infants from women with perinatal loss are more susceptible to independently rated suboptimal cognitive development compared to mother–infant dyads without perinatal loss. Assessing the nature and meaning of the perinatal loss is helpful when assessing infant development, as children born of women of perinatal loss are more vulnerable to poorer cognitive developmental outcomes. The results also provide rationale for increased support during pregnancy and infancy for women with previous perinatal loss to, in turn, facilitate more optimal infant cognitive development. There may also be potential utility in therapists providing targeted interventions for women with perinatal loss throughout the infancy and toddler period to support these mothers to provide a conducive environment for their children to developmentally thrive in. Further research is required to explore interventions that may support more favourable maternal attitudes and mother–infant interactions with women with perinatal loss.

## Figures and Tables

**Table 1:** Descriptive statistics and chi-square results of categorical variables comparing women with and without perinatal loss, N = 40.

Variable	Women with Perinatal Loss		Women without Perinatal Loss		χ^2^
	*n*	%	*n*	%	
**Marital status**	15	100.00	23	92.00	1.64
In a relationship	14	93.33	23	100.00	
Not in a relationship	1	6.67	0	0.00	
**Maternal ethnicity**	15	100.00	23	92.00	0.91
Caucasian	14	93.33	19	82.61	
Not Caucasian	1	6.67	4	17.39	
**Maternal mental health diagnosis**	15	100.00	22	88.00	0.04
Never	12	80.00	17	77.27	
Current or history of diagnosis	3	20.00	5	22.72	
**Infant gender**	15	100.00	25	100.00	0.03
Male	8	53.33	14	56.00	
Female	7	46.67	11	44.00	

χ2 = chi-square statistic. Total numbers in each variable may not equate to the totals for each sample due to missing data.

**Table 2:** Descriptive statistics of continuous variables for women with and without perinatal loss, N = 40.

	Women with Perinatal Loss (*n* = 15)						Women without Perinatal Loss (*n* = 25)							
Variable	*n*	%	Mean	SD	Min	Max	*n*	%	Mean	SD	Min	Max	*t*	α
Mother’s age (years)	15	100.00	30.07	5.23	19.00	38.00	23	92.00	29.83	3.56	23	37	0.17	
Other living children	15	100.00	0.00	0.00	0.00	0.00	22	88.00	0.36	0.73	0.00	3.00	−2.35 *	
Socio-economic status ^~^	15	100.00	73.80	21.01	33.00	100.00	23	92.00	76.26	16.13	25.00	98.00	−0.41	
Time 1 weeks’ gestation	13	86.67	29.15	1.68	27.00	33.00	23	92.00	32.35	7.22	13.00	41.00	−2.09	
Time 2 infant age (months)	15	100.00	18.20	3.95	12.00	25.00	25	100.00	19.60	2.90	12.00	25.00	0.71	
MHI-38 Mental Health Index	13	86.67	176.15	16.60	138.00	196.00	22	88.00	187.05	22.52	112.00	218.00	−1.51	0.95
**MAAS**														
Quality of att	12	80.00	49.50	3.63	44.00	55.00	23	92.00	51.35	3.16	43.00	54.00	−1.56	0.77
Time spent in att	13	86.67	27.62	5.41	18.00	35.00	23	92.00	28.70	4.60	20.00	37.00	−0.64	0.76
**ASQ**														
Att security	12	80.00	37.50	4.95	29.00	44.00	23	92.00	36.91	4.63	28.00	44.00	0.35	0.77
Att avoidance	12	80.00	42.83	9.68	31.00	58.00	23	92.00	44.57	10.81	27.00	65.00	−0.47	0.88
Att anxiety	12	80.00	44.75	9.21	32.00	62.00	23	92.00	44.52	11.31	26.00	64.00	0.06	0.88
**Bayley-III**														
Social-emotional	13	86.67	48.62	27.89	9.00	99.00	23	92.00	55.70	26.11	5.00	99.00	−0.76	
Adaptive behav	13	86.67	46.92	19.05	16.00	90.00	23	92.00	50.87	27.98	7.00	95.00	−0.45	
Cognitive	14	93.33	53.79	17.98	25.00	91.00	25	100.00	65.84	17.70	25.00	84.00	−2.02 *	
Language	14	93.33	42.29	18.27	23.00	79.00	25	100.00	53.84	20.75	23.00	92.00	−1.74	
Motor	14	93.33	46.14	23.62	16.00	88.00	25	100.00	59.76	19.62	21.00	98.00	−1.93	

* *p* ≤ 0.05. *t* = independent sample *t*-tests *t*-statistic; α = Cronbach’s alpha; ~ = based on Index of Relative Socio-economic Advantage and Disadvantage (IRSAD) percentile rank within Australia; MHI-38 = Mental Health Inventory-38; MAAS = Maternal Antenatal Attachment Scale; ASQ = Attachment Styles Questionnaire; Bayley-III = Bayley Scales of Infant and Toddler Development—Third Edition; att = attachment; behav = behavior. Total numbers in each variable may not equate to the totals for each sample due to missing data.

**Table 3:** Pearson correlation matrix of independent variables and infant developmental outcomes measured by Bayley-III, N = 40.

	Prenatal Maternal Mental Health	Quality of Pre Att	Time Spent in Prenatal Attachment	Maternal-Report		Observer-Rated		
				Social-Emotional	Adaptive Behavior	Cognition	Language	Motor
Perinatal loss	0.26	0.26	−0.08	0.13	0.08	0.32 *	0.28	0.30
Pre mental health	-	0.60 **	0.09	0.18	0.51 **	−0.19	−0.11	0.05
**MFA**								
Quality of att		-	0.48 **	0.11	0.40 *	0.03	−0.07	0.27
Time spent in att	0.05		-	0.12	0.01	−0.14	0.06	−0.10
**Adult attachment**								
Security	0.58 **	0.32	−0.06	0.17	0.52 **	0.17	0.32	0.23
Avoidance	−0.49 **	−0.33	−0.01	−0.15	−0.31	0.08	−0.13	−0.07
Att Anxiety	−0.54 **	−0.21	−0.01	−0.20	−0.49 **	−0.06	−0.13	−0.18

* *p* ≤ 0.05, ** *p* ≤ 0.01. MFA = maternal–fetal attachment; att = attachment; pre = prenatal.

**Table 4:** Multiple multivariate linear regression models with Bayley-III adaptive behavior as outcome variable and perinatal loss as control variable.

	Adaptive Behavior				
	B	S.E.	β	Adj R^2^	F
**Prenatal maternal mental health**	0.59	0.19	0.51 **	21.31	5.20 *
Perinatal loss	0.57	8.66	0.01		
**Quality of prenatal attachment**	3.11	1.33	0.41 *	10.58	2.83
Perinatal loss	−1.31	9.71	−0.02		
**Time spent in attachment**	0.03	0.96	0.01	-5.49	0.17
Perinatal loss	5.67	9.84	0.11		
**Attachment security**	3.18	1.02	0.54 **	23.17	4.92 *
Perinatal loss	2.67	10.00	0.05		
**Attachment avoidance**	−0.87	0.52	−0.33	3.15	1.42
Perinatal loss	2.29	11.24	0.04		
**Attachment anxiety**	−1.26	0.47	−0.48 *	16.77	3.62 *
Perinatal loss	−0.63	10.43	−0.01		

* *p* ≤ 0.05, ** *p* ≤ 0.01. B = Unstandardized beta, S.E. = Standard error, β = Standardized beta, Adj R^2^ = Adjusted R Square, F = F-test.

## Data Availability

The data presented in this study are available on request from the corresponding author. The data are not publicly available due to ethical reasons.
